# T2’-Imaging to Assess Cerebral Oxygen Extraction Fraction in Carotid Occlusive Disease: Influence of Cerebral Autoregulation and Cerebral Blood Volume

**DOI:** 10.1371/journal.pone.0161408

**Published:** 2016-08-25

**Authors:** Alexander Seiler, Ralf Deichmann, Waltraud Pfeilschifter, Elke Hattingen, Oliver C. Singer, Marlies Wagner

**Affiliations:** 1 Department of Neurology, Goethe University Frankfurt, Frankfurt, Germany; 2 Brain Imaging Center, Goethe University Frankfurt, Frankfurt, Germany; 3 Department of Neuroradiology, Rheinische Friedrich-Wilhelms-Universität Bonn, Bonn, Germany; 4 Department of Neurology, Helios HSK Hospital, Wiesbaden, Germany; 5 Institute of Neuroradiology, Goethe University Frankfurt, Frankfurt, Germany; Henry Ford Health System, UNITED STATES

## Abstract

**Purpose:**

Quantitative T2'-mapping detects regional changes of the relation of oxygenated and deoxygenated hemoglobin (Hb) by using their different magnetic properties in gradient echo imaging and might therefore be a surrogate marker of increased oxygen extraction fraction (OEF) in cerebral hypoperfusion. Since elevations of cerebral blood volume (CBV) with consecutive accumulation of Hb might also increase the fraction of deoxygenated Hb and, through this, decrease the T2’-values in these patients we evaluated the relationship between T2’-values and CBV in patients with unilateral high-grade large-artery stenosis.

**Materials and Methods:**

Data from 16 patients (13 male, 3 female; mean age 53 years) with unilateral symptomatic or asymptomatic high-grade internal carotid artery (ICA) or middle cerebral artery (MCA) stenosis/occlusion were analyzed. MRI included perfusion-weighted imaging and high-resolution T2’-mapping. Representative relative (r)CBV-values were analyzed in areas of decreased T2’ with different degrees of perfusion delay and compared to corresponding contralateral areas.

**Results:**

No significant elevations in cerebral rCBV were detected within areas with significantly decreased T2’-values. In contrast, rCBV was significantly decreased (p<0.05) in regions with severe perfusion delay and decreased T2’. Furthermore, no significant correlation between T2’- and rCBV-values was found.

**Conclusions:**

rCBV is not significantly increased in areas of decreased T2’ and in areas of restricted perfusion in patients with unilateral high-grade stenosis. Therefore, T2’ should only be influenced by changes of oxygen metabolism, regarding our patient collective especially by an increase of the OEF. T2’-mapping is suitable to detect altered oxygen consumption in chronic cerebrovascular disease.

## Introduction

Cerebral perfusion abnormalities are suspected to play a key role in the pathophysiology of carotid occlusive disease [1] and are believed to be strongly linked to embolic phenomena and stroke risk. [2] Hemodynamic impairment for the risk stratification of subsequent cerebral ischemia in patients with significant stenosis can be detected by the means of magnetic resonance imaging (MRI) using perfusion-weighted imaging (PWI). However, the association between the presence of perfusion disturbances alone and stroke risk is weak and perfusion abnormalities appear frequently in asymptomatic patients. [3] To account for this, other imaging techniques complementing conventional perfusion-weighted imaging with additional information on cerebral metabolic changes would be desirable.

Since an increased cerebral oxygen extraction fraction (OEF) has been shown to be an indicator of exhausted autoregulatory reserve and an independent risk factor for stroke in several longitudinal positron emission tomography (PET) studies on patients with unilateral high-grade carotid stenosis/occlusion, [4, 5] MRI techniques focusing on changes in cerebral oxygen metabolism seem to be a promising tool for the diagnostic workup of hemodynamically relevant stenosis.

Quantitative T2’-mapping has been used to describe changes in cerebral oxygen metabolism by using the different magnetic properties of oxygenated and deoxygenated hemoglobin (Hb). [6, 7] In contrast to diamagnetic oxygenated Hb, paramagnetic deoxygenated Hb decreases the relaxation time T2* of an imaging voxel. In contrast to T2*, T2’ (1/T2’ = 1/T2* - 1/T2) is corrected for changes in T2 relaxation time that are known to occur increasingly with age and in most cerebral pathologies. [8, 9] T2’ therefore allows for a more accurate estimation of changes in oxygen metabolism. [6]

T2’ has been shown to be decreased in perfusion-restricted brain regions in chronic cerebral hypoperfusion [10] as well as in acute ischemic stroke [11, 12] which was interpreted as a sign of increased OEF. However, a decrease of T2’, suggesting a shift in the relation between oxygenated and deoxygenated Hb in favor of deoxygenated Hb, can be the consequence of a decreased O_2_ supply at constant O_2_ demand, or can result from an increased O_2_ consumption and constant supply in case of neuronal activation. Further, an increased cerebral blood volume (CBV) might lead to an accumulation of deoxygenated Hb. The latter should be considered especially in patients with hemodynamically relevant stenosis, because cerebral autoregulation might try to compensate decreased cerebral blood flow (CBF) by an increase of CBV. [5]

In a previous study on patients with high grade occlusive carotid disease, [10] we found T2’ to be decreased in areas of restricted perfusion, which was interpreted as the result of an increased OEF only. However, the influence of CBV was not tested. We therefore sought to reinvestigate the potential influence of CBV-changes on the T2’-decrease within perfusion-restricted cerebral tissue in our patient collective [10] with unilateral high-grade occlusive carotid disease to validate the significance of our previous findings.

## Materials and Methods

We performed a retrospective analysis of a prospectively acquired dataset (approved by the local ethics committee of the Frankfurt University Hospital, Frankfurt, Germany) of which parts have previously been published by our group. [10] All patients had given written informed consent before the inclusion in the study.

### Patients

Imaging data including T2’-maps and CBV-maps, MTT- and TTP-maps of 16 patients (13 men; mean age ± SD, 54 ± 12.3 years, range 28–75 years) were analyzed. The patient characteristics, inclusion and exclusion criteria and perfusion deficits have been described in detail elsewhere. [10] Patients with the following vascular pathologies were included: unilateral symptomatic or asymptomatic 1) high-grade (≥70% according to the North American Symptomatic Carotid Endarterectomy Trial [NASCET] criteria) stenosis or occlusion of the extracranial internal carotid artery (ICA) verified by Doppler/Ultrasound or 2) high-grade (>50%) stenosis or occlusion of the intracranial ICA or middle cerebral artery (MCA) verified by MR-angiography and/or Doppler/Ultrasound. In several patients (n = 9), data from conventional digital subtraction angiography (DSA) was available for graduation of stenosis. We used these data to validate the degree of stenosis estimated from MR-angiography and Doppler/Ultrasound.

### MR imaging protocol

Data acquisition and postprocessing have been described in detail in several recent studies. [6, 10, 12] In brief, MR imaging data were acquired on a 3-T whole-body scanner (Trio, Magnetom Series; Siemens, Erlangen/Germany) equipped with a body transmit coil and an 8-channel phased array head receive coil. T2-maps were acquired using a turbo spin echo (TSE) sequence with the following imaging parameters: 50 axial slices with 2-mm slice thickness, no interslice gap, TR: 10 seconds, bandwidth: 100 Hz/pixel, 180° refocusing pulses, matrix size 192×132 (readout×phase encoding), field of view (FOV) 240×165 mm^2^, isotropic in-plane resolution 1.25 mm, turbo factor (number of spin echoes per excitation): 11, delay between subsequent echoes: 17.1 ms. For quantitative T2-mapping, five TSE data sets were acquired with identical parameters but different TE values (17, 86, 103, 120, 188 ms). The total duration for all five TSE data sets was 11 minutes and 50 seconds.

For high-resolution and motion-corrected [13] T2*-mapping, a series of T2*-weighted images with increasing TE was acquired, using a fast gradient echo (GE) sequence with the acquisition of eight GE per excitation pulse via successive inversion of the readout gradient, with data acquisition during positive polarity, only. The imaging parameters were: linear TE increase from 10 ms to 52 ms with a constant increment of 6 ms, TR: 3000 ms, 50 axial slices with 2-mm slice thickness, no interslice gap, bandwidth: 300 Hz/pixel, flip angle: 30°, matrix size 160×128 (readout×phase encoding), field of view 200×160 mm^2^, isotropic in-plane resolution 1.25 mm, duration 6 minutes 24 seconds. As quantitative T2*-mapping is considerably prone to artifacts induced by intrascan subject motion, the acquisition was repeated with half the spatial resolution in phase encoding direction (duration: 3 minutes 12 seconds). The k-space data of the full and the half resolution data sets were subsequently combined in a special manner for suppression of motion artefacts, as described earlier. [13]

Perfusion-weighted imaging was based on a GE echo-planar imaging (EPI) sequence (TE: 35 ms, TR: 1500 ms, flip angle: 90°, field of view 192×192 mm^2^, matrix size 64×64, isotropic in-plane resolution 2 mm, slice thickness 4 mm, number of slices 16, duration 75 seconds). For verification of intracranial stenosis and exclusion of significant (>50%) contralateral intracranial ICA or MCA stenosis, a 3-dimensional time-of-flight angiography was performed.

### Processing of MRI raw images

Raw images (turbo spin echo and perfusion-weighted) were linearly coregistered to the first raw T2*-weighted image by means of the FMRIB Linear Registration Tool (FLIRT–part of FSL, http://www.fmrib.ox.ac.uk/). Maps of T2 and T2* were derived by mono-exponential fitting of the respective data sets across the different TE values. Maps of T2′ were calculated as 1/T2′ = 1/T2*−1/T2.

Perfusion-weighted MRI raw images were processed on a pixel-by-pixel basis to generate maps of the time-to-peak (TTP), relative mean transit time (rMTT) and relative CBV (rCBV). The shape of the arterial input function was determined by manually choosing 5 to 10 pixels over the distal MCA trunk in the unaffected hemisphere. [14–16] For calculation of MTT- and rCBV-maps we used the model-independent (nonparametric) singular value decomposition deconvolution method described by Ostergaard et al. [17, 18]

### Data evaluation for artefact minimization

For the acquisition of quantitative T2- and T2*- maps, five spin echo and eight gradient echo data sets, respectively, were aquired at different echo times with a relatively high in-plane resolution that enabled a precise alignment for coregistration and for the calculation of T2’-maps. Due to the relatively long acquisition time however, this could only be achieved at the expense of an increased vulnerability to artifacts potentially induced by patients’ intrascan movement. Apart from our previously described motion correction algorithm, [13] this was accounted for by performing a thorough inspection of the T2*-weighted raw data for artefacts within the MCA territory. Afterwards, fit correlation maps depicting a dimension-less correlation coefficient (0–1.0) between the measured and fitted data were investigated for irregularities within the target regions. In order to exclude voxels with erroneous T2’-values and strongly motion-affected data, only voxels with a very high correlation coefficient were taken into consideration for further analysis.

### Region-of-interest definition and analysis

**Fig 1:**
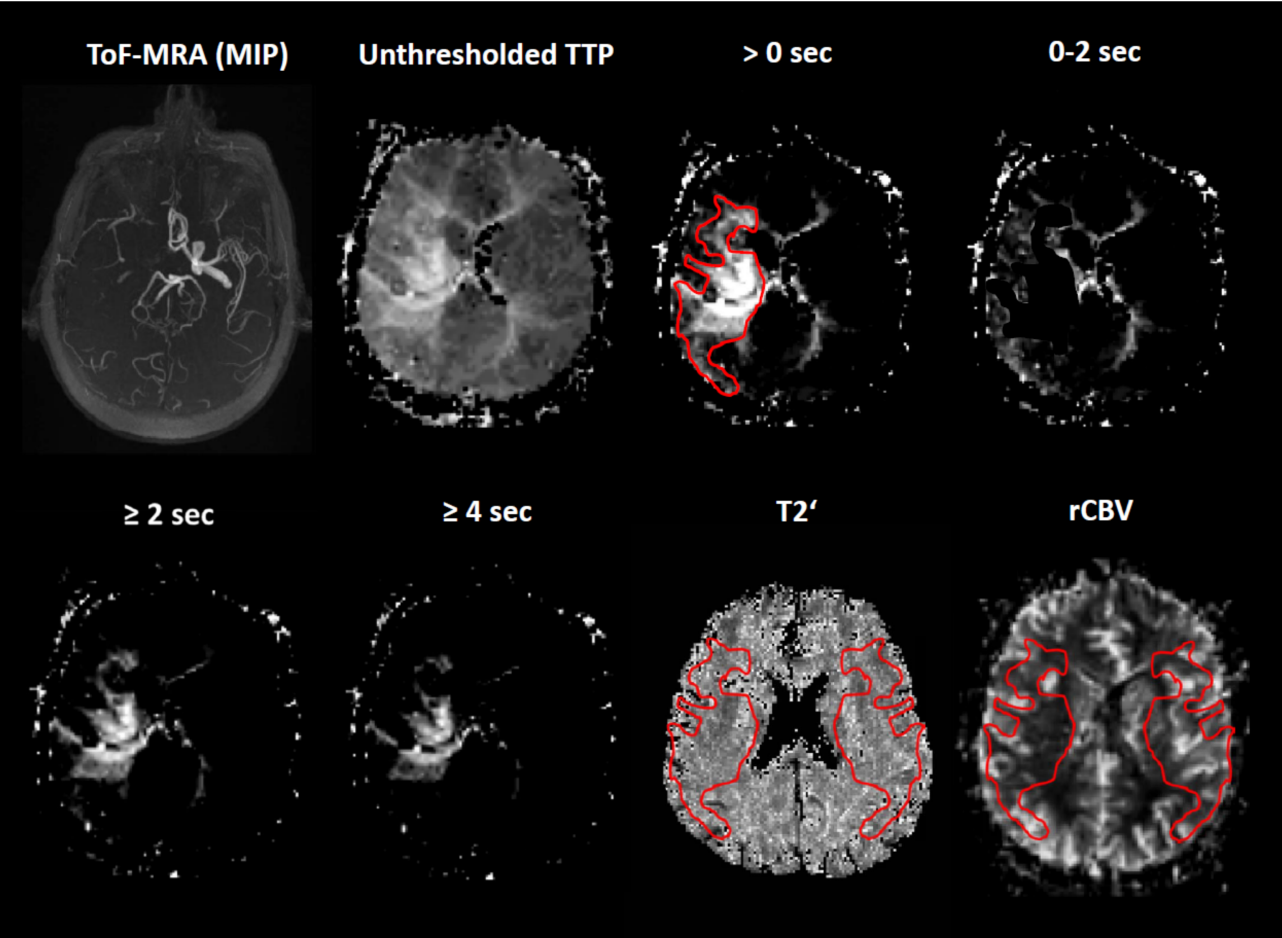
Description of ROI definition in a representative patient. MR-angiography shows no flow in the right intracranial ICA and MCA. TTP-images were thresholded according to different degrees of perfusion delay and perfusion delay ranges as compared to the contralateral unaffected hemisphere. The threshold >0 seconds represents the entire perfusion-disturbed area in relation to the upper limit of normoperfusion in the unaffected hemisphere. Areas of perfusion delay were manually outlined in order to generate ROIs which were then transferred to the coregistered T2’- and rCBV-maps. In order to quantify T2’ and rCBV in corresponding areas of the unaffected hemisphere, ROIs were mirrored to the contralateral side. MTT-maps were processed accordingly. ToF: time-of-flight; MRA: MR-angiography; MIP: Maximum Intensity Projection; TTP: time-to-peak; sec: seconds; rCBV: relative cerebral blood volume.

As time-to-peak (TTP) and mean transit time (MTT) are known to show substantial intersubject variation we used a semiquantitative method for the definition of perfusion abnormalities. Perfusion-disturbed areas on TTP- and MTT-maps were defined as regions with a perfusion delay in comparison to the upper limit of normoperfusion in the unaffected hemisphere, which we defined as the mean TTP and mean MTT plus two standard deviations. Mean TTP and MTT were assessed by placing a standardized circular region-of-interest (ROI, size 1170 mm²) in the MCA territory of the healthy hemisphere. First, the images were then thresholded according to the mean value plus two standard deviations which led to a delineation of the entire perfusion-restricted areas on TTP- and MTT-maps (perfusion delay > 0 sec). Furthermore, we gradually increased this threshold to receive areas with different perfusion thresholds (e.g. ≥2 sec, ≥ 4 sec, ≥6 sec, up to the maximum of perfusion delay) and perfusion delay ranges (0–2 sec, 2–4 sec, 4–6 sec, 6–8 sec delay) and for each patient (Fig 1). The latter was done for a more distinct delineation of rather slightly perfusion-disturbed areas. The margins for the perfusion delay ROIs could be delineated after thresholding. These ROIs were then subtracted from another in order to obtain the intervals (e.g. 0–2 s delay, Fig 1) which were used as ROIs for perfusion delay ranges. Since artefacts in gradient echo imaging due to field inhomogenities can especially be expected near the paranasal sinuses and remote cortical areas we chose several adjacent slices on the level of the lateral ventricles to generate three-dimensional ROIs. Areas of perfusion delay were outlined manually and transferred to the coregistered T2’- and rCBV-maps. To compare T2’- and rCBV-values within perfusion-restricted areas with corresponding values in contralateral healthy tissue the ROIs were mirrored to the contralateral side which enabled an exact assessment of T2’ and rCBV in corresponding normoperfused areas (Fig 1). The use of TTP- and MTT-masks allowed for analysis of rCBV at different stages of perfusion delay. Due to the wide scattering of T2’-values [10], the use of thresholded T2’-based masks was not reasonable. T2’- and rCBV-values for the corresponding ROIs were extracted to calculate median values over all selected slices. Since both T2'- and rCBV-values were expected to show considerable interindividual variation, we also calculated hemispheric ratios by dividing values obtained from perfusion-restricted tissue by values from corresponding contralateral areas. To account for the poor signal-to-noise ratio of T2'-maps and to minimize an impact of zero voxels and unplausibly high T2'-values (e.g. voxels containing CSF) on the median value of each ROI, the maps were thresholded at ≥1 ms (lower threshold) and ≤300 ms (upper threshold). ROI-placement and analysis was done with the Java-based software ImageJ (http://rsb.info.nih.gov/ij/index.html). Moreover, we calculated R2’ (1/T2’)-values since R2’ is assumed to be proportional to rCBV and OEF [19] and might therefore be useful to evaluate the direct influence of rCBV changes on oxygen-sensitive MR imaging sequences.

### Statistical analysis

As both T2’- and rCBV-values were not normally distributed we only performed non-parametric statistical testing: the Wilcoxon-signed rank test was used for comparison of T2’, R2’ and rCBV within perfusion-restricted and contralateral areas, the Spearman’s rank correlation for the assessment of a parameter correlation and the Mann-Whitney test was used for group comparisons. Significance level was set to p<0.05. Statistical analysis was performed with SPSS 22 (IBM SPSS Statistics, SPSS Inc). Due to the skewed distribution data are given as median (25^th^, 75^th^ percentile) throughout the results section unless stated otherwise.

## Results

### rCBV in areas of decreased T2’ and in perfusion-restricted tissue

**Fig 2:**
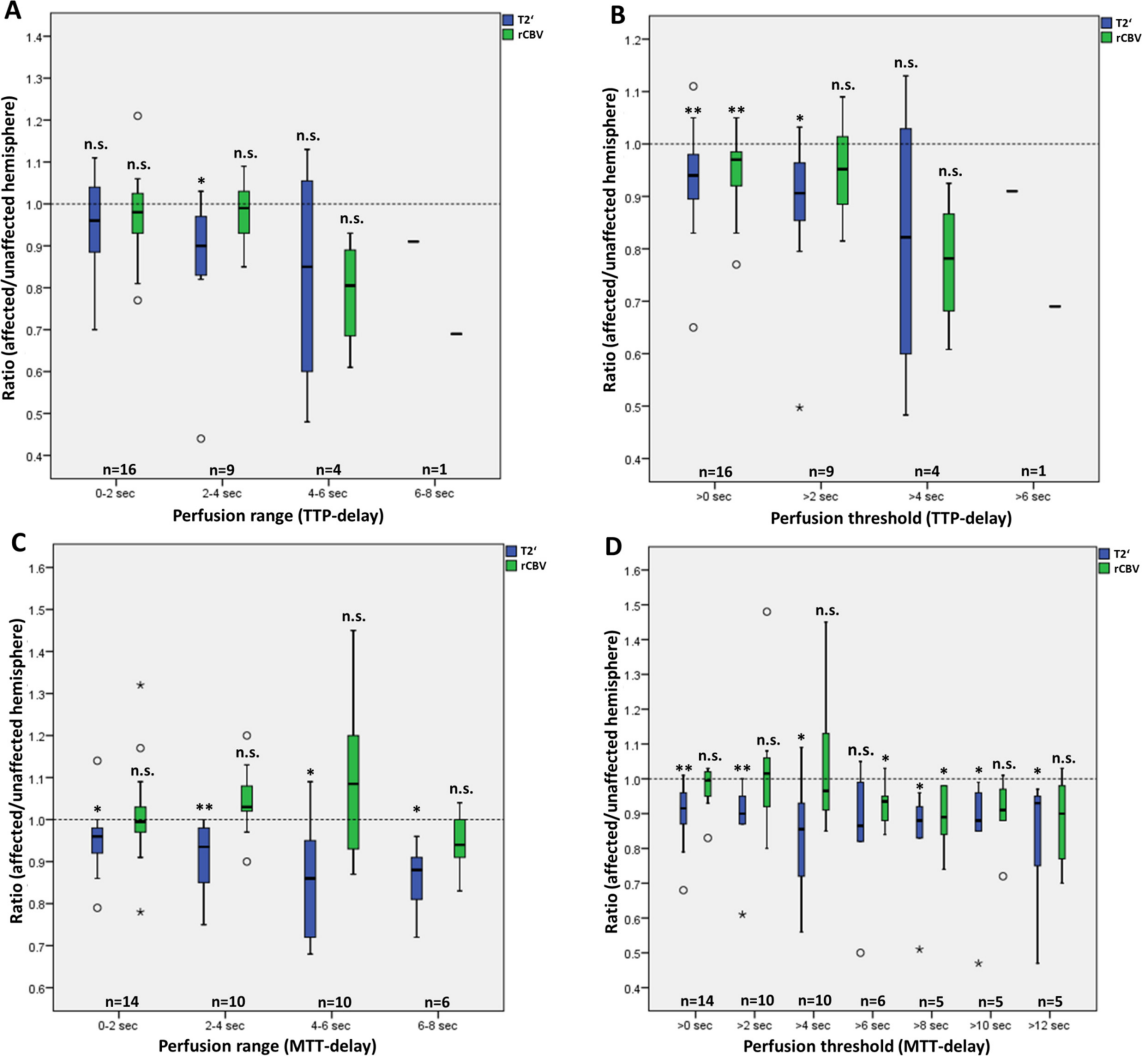
**Boxplots of median hemispheric ratios of T2’ and rCBV for areas with different degrees of perfusion delay ranges and perfusion delays on TTP- (A, B) and MTT- (C, D) maps.** Asterisks above the boxplots indicate significant changes of T2’ and rCBV as compared to contralateral unaffected tissue. *: p<0.05; **: p< 0.01; n.s.: not significant; sec: seconds; TTP: time-to-peak; MTT: mean transit time; rCBV: relative cerebral blood volume.

rCBV did not show a significant increase in areas of decreased T2’. When measured in areas with different degrees of perfusion delay, rCBV showed significant changes only in areas of any TTP-delay (>0 s) and of a MTT-delay ≥6 s and ≥8 s, where rCBV was decreased (p = 0.006, p = 0.046, p = 0.043). In these areas (except for an MTT-delay ≥6 s), T2’ was also significantly (p = 0.008, p = 0.043) decreased (Table 1 for details, Fig 2B and 2D for a graphic overview). Besides, a significant increase of rCBV could not be found in any area of impaired perfusion (Table 1). Although not statistically significant, rCBV showed to be increased in areas of slight to moderate MTT-delay (2–4 s, 4–6 s), while it showed to decrease with further increasing MTT-delay (6–8 s, Fig 2C). This trend was not comprehensible in areas of different TTP-delays (Table 1, Fig 2A and 2B). The analysis of R2’-values in areas showed very similar results in terms of significance, but with an increase of R2’ in perfusion-restricted areas (R2’ = 1/T2’) (Table 2).

**Table 1:** Values and hemispheric ratios of T2’ and rCBV for different degrees of perfusion delay in perfusion-disturbed and corresponding normoperfused tissue.

		Median T2’ [ms] (25^th,^ 75^th^ percentile)				Median rCBV [ml/100 g] (25^th^, 75^th^ percentile)			
		Affected hemisphere	Unaffected hemisphere	Ratio	P-value	Affected hemisphere	Unaffected hemisphere	Ratio	P-value
0 sec	TTP	118 (108.75, 120)	127.5 (116.75, 134.75)	0.93 (0.88, 0.99)	0.008	6.97 (4.5, 9.63)	7.18 (5.68, 9.7)	0.97 (0.92, 0.99)	0.006
	MTT	116 (106.5, 199.25)	131 (120.75, 135)	0.92 (0.87, 0.96)	0.002	7.12 (4,72, 9.14)	7.04 (5, 9.02)	1 (0.95, 1.03)	0.47
0–2 sec	TTP	118 (108.75, 122.25)	124 (114.5, 130.75)	0.96 (0.88, 1.05)	0.082	7.05 (4.4, 7.99)	7.28 (5.32, 8.34)	0.98 (0.93, 1.03)	0.278
	MTT	118 (111.5, 119.5)	122.5 (115.5, 131.25)	0.96 (0.91, 0.99)	0.018	7.41 (5.01, 9.92)	7.16 (5.09, 10.96)	1 (0.96, 1.05)	0.73
2–4 sec	TTP	121 (115.5, 128)	135 (125, 145)	0.90 (0.83, 1)	0.028	7.17 (5.67, 9.11)	7.36 (5.79, 9.62)	0.99 (0.92, 1.04)	0.374
	MTT	115.5 (106, 125.75)	129 (115.25, 139)	0.94 (0.83, 0.98)	0.008	7.69 (6.11, 12.99)	8.06 (5.52, 12.7)	1.03 (1.01, 1.09)	0.093
4–6 sec	TTP	120 (104.5, 137.75)	142.5 (126.5, 198.25)	0.85 (0.54, 1.09)	0.273	6.23 (2.53, 10.55)	7.90 (3.68, 12.24)	0.81 (0.65, 0.91)	0.068
	MTT	112.5 (110.25, 120.25)	132 (122.75, 143.25)	0.86 (0.71, 0.96)	0.015	7.96 (6.1, 16.57)	8.52 (5.25, 14.79)	1.09 (0.92, 1.25)	0.169
6–8 sec^a^	TTP	124	136	0.91	-----	8.22	11.92	0.69	-----
	MTT	126 (112.5, 136)	138 (131, 167)	0.88 (0.77, 0.94)	0.043	7.68 (6.85, 12.06)	8.47 (7.03, 13.04)	0.94 (0.87, 1.02)	0.138
≥ 2 sec	TTP	121 (112.5, 130)	135 (128, 141)	0.91 (0.83, 0.97)	0.013	6.84 (5.44, 8.77)	6.61 (6.14, 9.16)	0.95 (0.86, 1.02)	0.139
	MTT	114.5 (108.5, 121.5)	130 (118.6, 136.75)	0.90 (0.87, 0.96)	0.008	8.28 (5.91, 12.37)	7.14 (6.17, 10.61)	1.01 (0.92, 1.07)	0.721
≥ 4 sec	TTP	118 (104.5, 136.75)	144 (127.25, 198.25)	0.83 (0.54, 1.08)	0.273	7.23 (2.53, 9.83)	7.9 (3.68, 11.82)	0.79 (0.65, 0.9)	0.068
	MTT	112.5 (88.75, 118.5)	132 (123.75, 138)	0.86 (0.71, 0.95)	0.011	7.8 (5.9, 12.75)	8.29 (5.79, 12.84)	0.97 (0.91, 1.2)	0.799
≥ 6 sec^a^	TTP	124	136	0.91	-----	8.22	11.92	0.69	-----
	MTT	115 (101.75, 134)	135.5 (128, 141.75)	0.87 (0.74, 1.01)	0.075	7.18 (6.58, 9.85)	7.71 (6.82, 10.71)	0.94 (0.87, 0.97)	0.046
≥ 8 sec	MTT	113 (95.5, 126.5)	137 (128.5, 147.5)	0.88 (0.67, 0.94)	0.043	7.32 (6.38, 9.83)	8.68 (6.77, 11.79)	0.89 (0.79, 0.98)	0.043
≥ 10 sec	MTT	116 (95, 127)	137 (123.5, 153)	0.88 (0.66, 0.98)	0.043	7.88 (6.02, 9.34)	8.64 (6,37, 11.39)	0.91 (0.8, 0.99)	0.08
≥ 12 sec	MTT	115 (100.5, 128)	139 (124.5, 172)	0.93 (0.61, 0.96)	0.043	8.34 (5.22, 9.92)	8.53 (5.89, 12.57)	0.9 (0.74, 1.01)	0.08

Table 1. ms: milliseconds; ml: milliliters; g: grams; sec: seconds; TTP: time-to-peak; MTT: mean transit time.

^a^Only 1 patient showed a TTP-delay of ≥6 and 6–8 seconds. Therefore, no percentiles and p-values could be determined.

**Table 2:** Values and hemispheric ratios of R2’ und rCBV for different degrees of perfusion delay in perfusion-disturbed and corresponding normoperfused tissue.

		Median R2’ [s^-1^] (25^th,^ 75^th^ percentile)				Median rCBV [ml/100 g] (25^th^, 75^th^ percentile)			
		Affected hemisphere	Unaffected hemisphere	Ratio	P-value	Affected hemisphere	Unaffected hemisphere	Ratio	P-value
0 sec	TTP	8.47 (8.33, 9.20)	7.84 (7.42, 8.57)	1.07 (1.02, 1.13)	0.011	6.97 (4.5, 9.63)	7.18 (5.68, 9.7)	0.97 (0.92, 0.99)	0.006
	MTT	8.63 (8.38, 9.39)	7.64 (7.41, 8.29)	1.09 (1.04, 1.15)	0.002	7.12 (4,72, 9.14)	7.04 (5, 9.02)	1 (0.95, 1.03)	0.47
0–2 sec	TTP	8.47 (8.18, 9.20)	8.07 (7.65, 8.74)	1.04 (0.96, 1.13)	0.088	7.05 (4.4, 7.99)	7.28 (5.32, 8.34)	0.98 (0.93, 1.03)	0.278
	MTT	8.47 (8.37, 8.97)	8.17 (7.62, 8.66)	1.04 (1.02, 1.10)	0.023	7.41 (5.01, 9.92)	7.16 (5.09, 10.96)	1 (0.96, 1.05)	0.73
2–4 sec	TTP	8.26 (7.81, 8.67)	7.41 (6.90, 8.00)	1.12 (1.00, 1.21)	0.038	7.17 (5.67, 9.11)	7.36 (5.79, 9.62)	0.99 (0.92, 1.04)	0.374
	MTT	8.66 (7.95, 9.43)	7.76 (7.19,8.68)	1.07 (1.02, 1.20)	0.008	7.69 (6.11, 12.99)	8.06 (5.52, 12.7)	1.03 (1.01, 1.09)	0.093
4–6 sec	TTP	8.45 (7.26, 9.57)	7.03 (5.18, 7.92)	1.21 (0.92, 1.90)	0.273	6.23 (2.53, 10.55)	7.90 (3.68, 12.24)	0.81 (0.65, 0.91)	0.068
	MTT	8.89 (8.31, 10.03)	7.58 (6.99, 8.15)	1.16 (1.04, 1.41)	0.015	7.96 (6.1, 16.57)	8.52 (5.25, 14.79)	1.09 (0.92, 1.25)	0.169
6–8 sec^a^	TTP	8.06	7.35	1.10	-----	8.22	11.92	0.69	-----
	MTT	7.94 (7.37, 8.89)	7.25 (6.00, 7.64)	1.13 (1.07, 1.32)	0.043	7.68 (6.85, 12.06)	8.47 (7.03, 13.04)	0.94 (0.87, 1.02)	0.138
≥ 2 sec	TTP	8.26 (7.69, 8.89)	7.49 (7.09, 7.82)	1.10 (1.04, 1.13)	0.015	6.84 (5.44, 8.77)	6.61 (6.14, 9.16)	0.95 (0.86, 1.02)	0.139
	MTT	8.74 (8.23, 9.22)	7.70 (7.31, 8.44)	1.11 (1.04, 1.15)	0.008	8.28 (5.91, 12.37)	7.14 (6.17, 10.61)	1.01 (0.92, 1.07)	0.721
≥ 4 sec	TTP	8.56 (7.32, 9.57)	6.95 (5.18, 7.88)	1.24 (0.93, 1.90)	0.273	7.23 (2.53, 9.83)	7.9 (3.68, 11.82)	0.79 (0.65, 0.9)	0.068
	MTT	8.89 (8.44, 11.27)	7.58 (7.25, 8.08)	1.17 (1.06, 1.41)	0.013	7.8 (5.9, 12.75)	8.29 (5.79, 12.84)	0.97 (0.91, 1.2)	0.799
≥ 6 sec^a^	TTP	8.06	7.35	1.10	-----	8.22	11.92	0.69	-----
	MTT	8.71 (7.46, 10.00)	7.38 (7.09, 7.81)	1.16 (1.00, 1.41)	0.075	7.18 (6.58, 9.85)	7.71 (6.82, 10.71)	0.94 (0.87, 0.97)	0.046
≥ 8 sec	MTT	8.85 (7.92, 10.76)	7.30 (6.81, 7.80)	1.14 (1.06, 1.58)	0.043	7.32 (6.38, 9.83)	8.68 (6.77, 11.79)	0.89 (0.79, 0.98)	0.043
≥ 10 sec	MTT	8.62 (7.88, 10.84)	7.30 (6.59, 8.12)	1.13 (1.03, 1.64)	0.043	7.88 (6.02, 9.34)	8.64 (6,37, 11.39)	0.91 (0.8, 0.99)	0.08
≥ 12 sec	MTT	8.70 (7.81, 10.09)	7.19 (5.89, 8.05)	1.08 (1.04, 1.74)	0.043	8.34 (5.22, 9.92)	8.53 (5.89, 12.57)	0.9 (0.74, 1.01)	0.08

Table 2. s: seconds; ml: milliliters; g: grams; sec: seconds; TTP: time-to-peak; MTT: mean transit time.

^a^Only 1 patient showed a TTP-delay of ≥6 and 6–8 seconds. Therefore, no percentiles and p-values could be determined.

### Correlation of T2’- and rCBV-values

When correlating median T2’-values with hemispheric ratios of rCBV we found a not significant positive correlation for areas with TTP-delay (r = 0.129, p = 0.331) and a weak not significant negative correlation for areas with MTT-delay (r = -0.039, p = 0.699). Also the correlation of T2’ and rCBV hemispheric ratios did not reveal any significant relationship, neither for areas with TTP hypoperfusion (r = 0.086, p = 0.516) nor for areas with MTT-delay (r = 0.011, p = 0.913). Similar results were obtained when analyzing the correlation between R2’-values and R2’ hemispheric ratios with rCBV ratios (S1 Table). When comparing T2’-values between patients with elevated rCBV (n = 6) and normal or reduced rCBV (n = 8) in areas with any MTT-delay (> 0 seconds) we found no significant difference for absolute T2’-values (p = 1.000) and T2’ hemispheric ratios (p = 0.573) between the two groups. Individual patient data with baseline T2’- and rCBV-values are summarized in the S2 Table.

**Fig 3:**
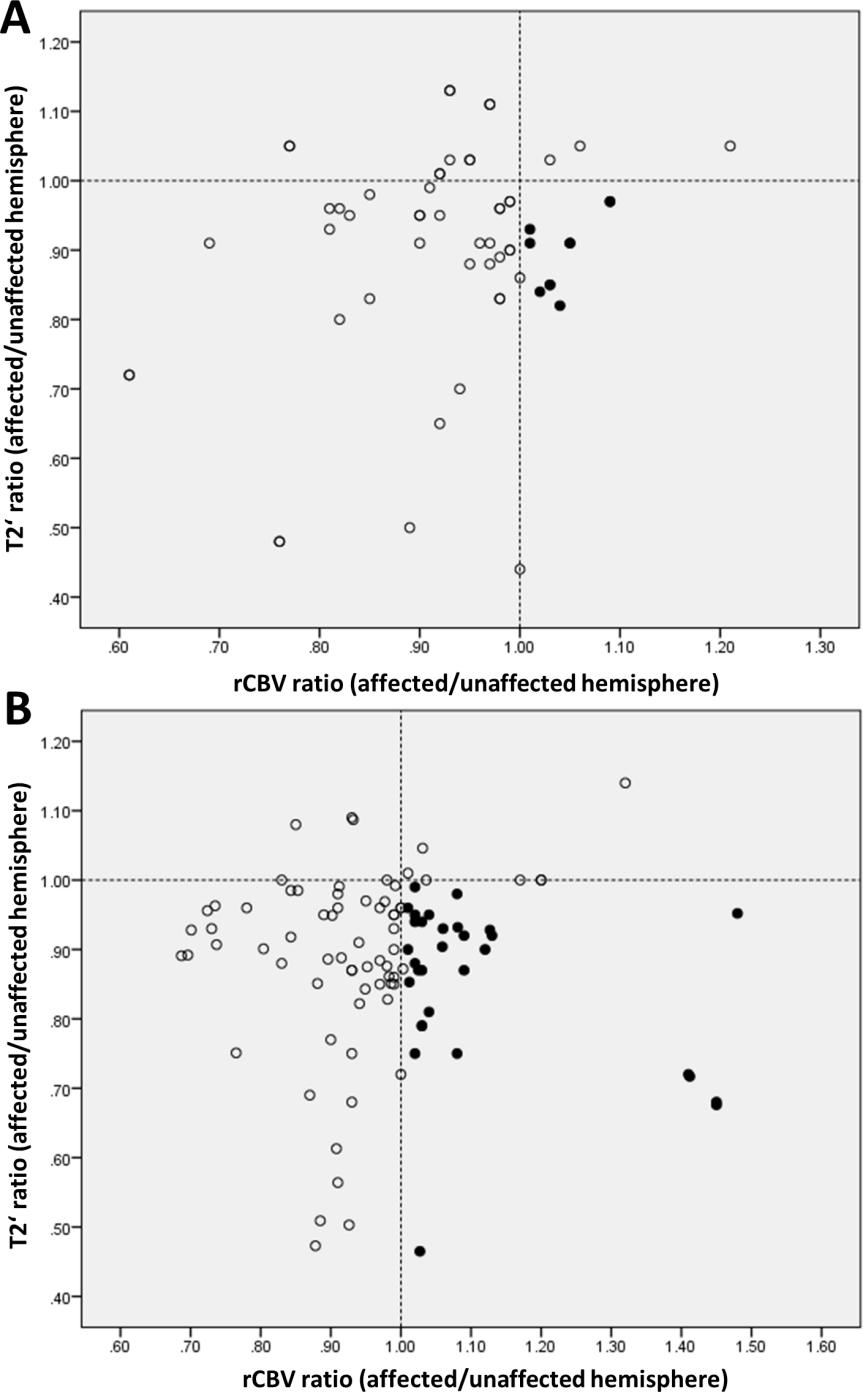
**Scatter plot of hemispheric ratios of T2’ and rCBV for all areas with TTP- (59 values calculated from 177 ROIs) (A) and MTT- (101 values calculated from 303 ROIs) (B) delay.** Broken lines indicate unity for T2’ and rCBV (hemispheric ratio = 1). Lower right quadrant (black dots) contains ROIs with reduced T2’ (hemispheric ratio<1) and simultaneously elevated rCBV (hemispheric ratio>1). rCBV: relative cerebral blood volume.

**Fig 4:**
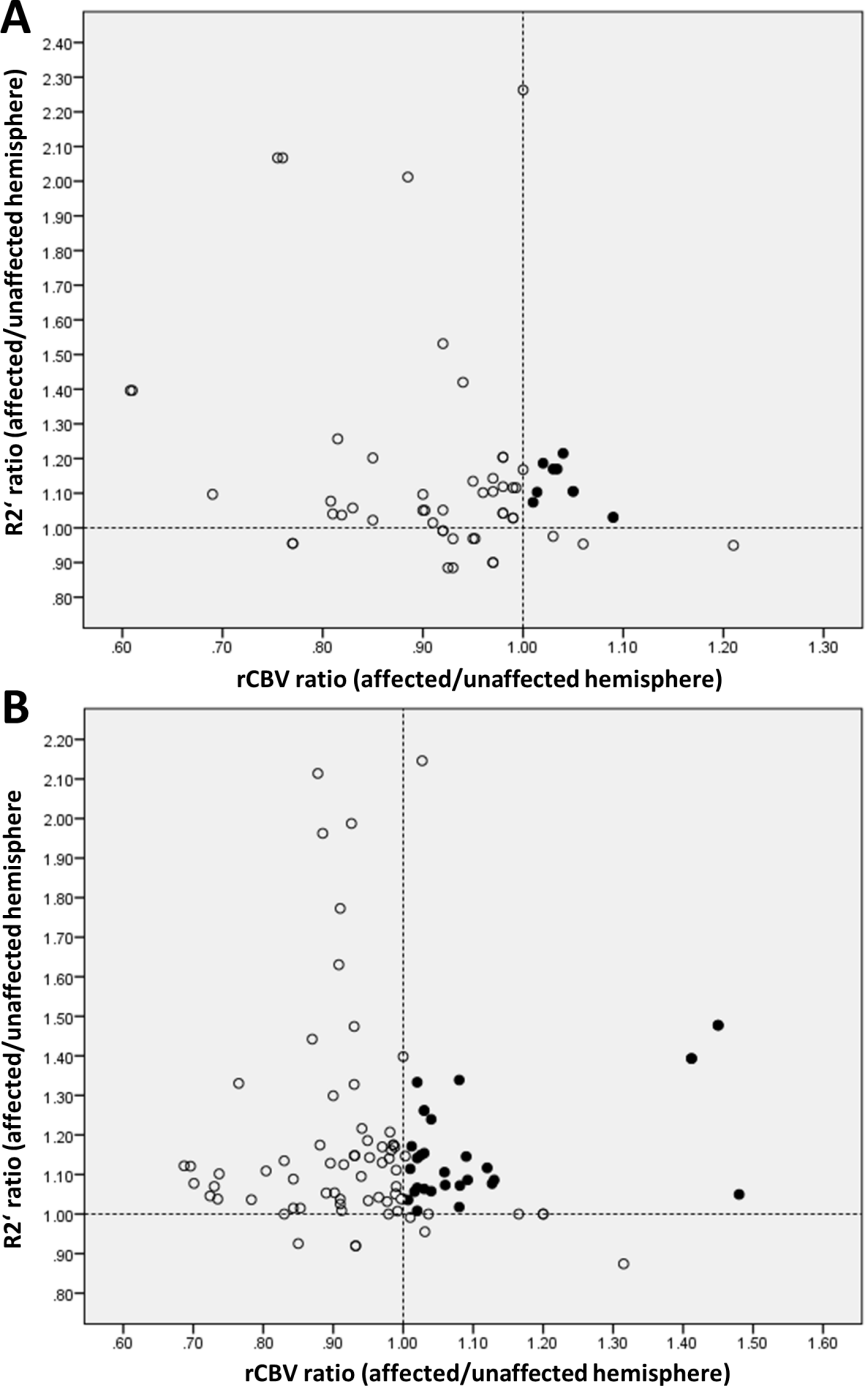
**Scatter plot of hemispheric ratios of R2’ and rCBV for all areas with TTP- (A) and MTT-delay (B).** Broken lines indicate unity of R2’ and rCBV in both hemispheres (hemispheric ratio = 1). Upper right quadrant (black dots) contains ROIs with increased R2’ (hemispheric ratio>1) and simultaneously elevated rCBV (hemispheric ratio>1). rCBV: relative cerebral blood volume.

Considering all ROIs in the analysis there were few ROIs in which we found a significantly reduced T2’ (respectively increased R2’) with simultaneously elevated rCBV (hemispheric ratio>1) (Figs 3 and 4A and 4B). The coincidence of these parameter changes was mainly restricted to regions with slight to moderate perfusion delay.

## Discussion

Our study underlines the validity of T2’ to detect changes of cerebral oxygen metabolism in patients with chronic hypoperfusion.

It is known that T2’ is decreased in areas of hypoperfusion in patients with unilateral high- grade ICA or MCA stenosis. [10] T2’ is considerably influenced by the relation of diamagnetic oxygenated to paramagnetic deoxygenated Hb, and an increasing concentration of deoxygenated Hb leads to a decrease of the relaxation time T2’. In patients with chronic hypoperfusion, a decrease of T2’ might be explained by an increase of the OEF to maintain constant oxygen consumption whilst reduced oxygen supply. Due to the cerebral autoregulation however that tries to compensate the decreased CBF by increasing the CBV, a decrease of T2’ might also be caused by an increasing CBV accumulating deoxygenated Hb.

We therefore tested possible changes of CBV in our previously published collective [10] of patients with unilateral high grade occlusive disease of the MCA or ICA that had shown significant decrease of T2’ in areas of impaired perfusion. We found that there is no correlation between rCBV and T2’. The decrease of T2’ is largely independent from alterations of rCBV since we found no significant increase of rCBV in areas of significantly decreased qT2’ and no significant correlation between changes of T2’ and changes of CBV. In detail, T2’-values were significantly decreased for all degrees of ipsilateral MTT-delay in patients with unilateral stenosis and showed further decrease with escalating perfusion delay [10] (Fig 2C and 2D). According to its calculation, MTT (MTT = CBV/CBF) functions as an index of autoregulatory reserve in cerebral hypoperfusion conditions. Prolonged MTT in hypoperfusion can therefore be caused either by a decrease of CBF accompanied by a stable or decreased CBV, which would lead to a less pronounced prolongation of MTT. In the other case, the decrease of CBF would be accompanied by a compensating increase of CBV, which would further increase the MTT-delay. In the light of the above, there are two major possible reasons for the finding of reduced T2’, suggesting an increased concentration of deoxygenated Hb in regions with prolonged MTT: 1. Increase in OEF due to a decreased O_2_ supply (CBF) at constant demand, which is expected to occur in case of an exhausted autoregulatory capacity. Decreased T2’ would then result primarily from the increased deoxygenated Hb concentration as a result of a preserved O_2_ consumption at decreased supply. 2. Elevations of rCBV with an accumulation of Hb as a consequence of autoregulatory vasodilation. In this case, the increasing accumulation of deoxygenated Hb in areas of increased CBV would considerably contribute to the decrease of T2’ in areas of prolonged MTT, and T2’ could not be used to reliably estimate the OEF.

We found that in areas of slight to moderate MTT-delay (2–4, 4–6, or ≥2 s), hemispheric ratios of rCBV indeed tend to increase up to 9%, but rCBV-values did not differ significantly between the perfusion-disturbed and the contralateral corresponding areas (Table 1 for details and Fig 2C and 2D for a graphic overview). According to the composition of total CBV reported in the literature, venous CBV amounts to 70–83% [20–22] of total CBV under normal physiologic conditions. However, the extent to which venous CBV contributes to increased total CBV in the case of autoregulatory vasodilation in hemodynamic impairment is unknown, and results of experimental animal studies are heterogeneous. [23, 24] We cannot totally exclude contributions of venous compartments with increased CBV to the reduced T2’-values. It is more likely however, that the relation between arterial and venous components is rather shifted to the arterial part as a result of reduced cerebral perfusion pressure. Increases–even minor—in arterial CBV as a result of autoregulatory dilation of arterial resistance vessels however should not contribute to changes in T2’ because the relation of oxygenated to deoxygenated Hb should be constant. This assumption further relativizes the slight increase of CBV with regard to its possible influence on T2’.

With increasing MTT-delay (6–8 s, ≥4 s) and in the entire TTP-delayed area (>0 s delay), hemispheric ratios of rCBV decreased up to 6% and rCBV-values also tended to decrease with a significant decrease in areas with MTT-delay ≥6 and ≥8 seconds compared to the contralateral hemisphere (Fig 2C and 2D). As T2’ decreased with increasing MTT-delay, those findings strongly support the hypothesis that T2’ can be used to estimate changes of OEF in patients with chronic hypoperfusion and that the decrease of T2’ is not influenced by an increased rCBV in those patients.

These findings are in line with the literature. [5, 25, 26] CBV largely depends on metabolic factors, and compensating changes of CBV and OEF are gradual processes. [25] In patients with regional hemodynamic failure, e.g. unilateral carotid stenosis, CBV is increased as long as an increased OEF is able to compensate CBF reduction. In areas of severe ischemia however, CBV is decreased as a consequence of hemodynamic failure. [25] Another explanation for CBV decrease is the so called ‘metabolic vasoconstriction’ [25] which occurs in areas of reduced metabolic activity. Chronic hypoperfusion is known to result in atrophic and gliotic changes of brain parenchyma which lead to a decreased metabolic demand compared to healthy neuronal parenchyma. [27, 28] One might assume that the slight CBV decrease in patients with severe MTT-delay is, at least in part, a result of degenerative changes of brain parenchyma due to the chronic hypoperfusion.

### Limitations

Our study has to deal with several limitations that should be addressed. CBV was measured by the means of dynamic susceptibility contrast (DSC) imaging. It has to be stated that those values are only representative relative values due to different influencing technical factors. [29] When compared to previous studies using dynamic susceptibility contrast MRI for the determination of age-dependent normal perfusion values of rCBV, median values for rCBV obtained in our study are slightly higher. [30–32] A conceivable reason for this finding might be fact that the vast majority of patients included in our study were male since rCBV in healthy male subjects is known to be significantly higher compared to female subjects. [31] In addition, the mean age of 53 years in our study population might contribute to the relatively high rCBV-values. CBV has been shown to increase with age, showing a remarkable elevation at the age of > 50 years compared to a younger population. [31] CBV shows large intersubject variation—particularly in patients with chronic perfusion abnormalities of different severities. However, the rCBV-values obtained in our study showed a good intra- and interindividual comparability. We therefore conclude that these values can be considered plausible.

Further, we did not differentiate between different compounds contributing to total CBV. Although arterial CBV should not have a relevant influence on T2’ as we assume a constant arterial blood oxygen saturation, for a more precise assessment of the relationship between T2’ and CBV venous and arterial proportions of CBV should be estimated separately. In addition, our sample size was small and we included symptomatic and asymptomatic patients that might differ in terms of risk profiles as well as hemodynamic and metabolic parameters. Apart from changes of oxygen metabolism, T2’- and R2’-values could be influenced by iron deposition in hypoperfused tissue, both due to pathologic changes of iron metabolism and blood-brain barrier damage. Although we found no evidence for this in our data, being able to rule out these possible conditions would make our results more reliable. Last, our single-center results require an external confirmation by others and a validation with the golden standard for the imaging of cerebral oxygen metabolism, PET.

## Conclusions

The findings of this study support the hypothesis that T2’ can be used to assess the grade of metabolic impairment in patients with unilateral high grade carotid stenosis. It could be shown that the decrease of T2’, presumably reflecting an increase of OEF, in the territory of a unilateral high-grade stenotic large-artery is largely independent from changes of CBV as measurable by MRI. Although giving only representative relative values for CBV, MRI with T2’ and DSC-perfusion can therefore be used reliably to assess changes of oxygen metabolism in patients with chronic cerebral hypoperfusion.

## Supporting Information

S1 File Values for T2’, R2’ and rCBV measured in areas with different degrees of time-to-peak-delay. (XLS)

S2 File Values for T2’, R2’ and rCBV measured in areas with different degrees of mean transit time-delay. (XLS)

S1 Table Correlation of R2’ (median, hemispheric ratios) and rCBV ratios in perfusion-restricted areas (Spearman’s rank correlation). PWI: perfusion-weighted imaging; TTP: time-to-peak; MTT: mean transit time.
(DOCX)

S2 Table Individual patient data: median values and hemispheric ratios for T2’ and rCBV in areas with any TTP- and MTT-delay (> 0 seconds). Due to the rather small differences in rCBV-values between the hemispheres, these data are presented with two decimals. In patient 5 and patient 14 no MTT-delay was detected. q: quantitative; rCBV: relative cerebral blood volume; TTP: time-to-peak; MTT: mean transit time. *Baseline absolute values of qT2’ for the entire TTP-and MTT-delayed areas have already been presented in a previous publication (Seiler A, Jurcoane A, Magerkurth J, Wagner M, Hattingen E, Deichmann R et al. T2' imaging within perfusion-restricted tissue in high-grade occlusive carotid disease. Stroke. 2012;43:1831–1836).
(DOCX)
