# Correction: T2-Imaging to Assess Cerebral Oxygen Extraction Fraction in Carotid Occlusive Disease: Influence of Cerebral Autoregulation and Cerebral Blood Volume

**DOI:** 10.1371/journal.pone.0164749

**Published:** 2016-10-12

**Authors:** The PLOS ONE Staff

## Notice of Republication

This article was republished on September 8, 2016, to correct an error in the article title. The phrase “T2-Imaging” should be “T2’-Imaging.” The publisher apologizes for the error. Please download this article again to view the correct version. The originally published, uncorrected article and the republished, corrected article are provided here for reference**.**

## Supporting Information

S1 File Originally published, uncorrected article. (PDF)

S2 File Republished, corrected article. (PDF)

## References

[1] Seiler A, Deichmann R, Pfeilschifter W, Hattingen E, Singer OC, Wagner M (2016) T2-Imaging to Assess Cerebral Oxygen Extraction Fraction in Carotid Occlusive Disease: Influence of Cerebral Autoregulation and Cerebral Blood Volume. PLoS ONE 11(8): e0161408 doi: 10.1371/journal.pone.0161408 27560515 10.1371/journal.pone.0161408 PMC4999181

