# Supplementary material for: T2’-Imaging to Assess Cerebral Oxygen Extraction Fraction in Carotid Occlusive Disease: Influence of Cerebral Autoregulation and Cerebral Blood Volume
Source: PLoS One. 2016 Aug 25;11(8):e0161408. doi: 10.1371/journal.pone.0161408 (PMC4999181; doi:10.1371/journal.pone.0161408)
Supplement: S1 Table — PWI: perfusion-weighted imaging; TTP: time-to-peak; MTT: mean transit time. (DOCX) [file pone.0161408.s003.docx]

| **PWI parameter** | **R2’** | **Correlation coefficient r** | **p-value** |
| --- | --- | --- | --- |
| **TTP** | **Median** | -0.145 | 0.273 |
|  | **Ratio** | -0.076 | 0.569 |
| **MTT** | **Median** | 0.04 | 0.695 |
|  | **Ratio** | -0.01 | 0.919 |

**S1 Table.** Correlation of R2’ (median, hemispheric ratios) and rCBV ratios in perfusion-restricted areas (Spearman’s rank correlation).
