# Supplementary material for: T2’-Imaging to Assess Cerebral Oxygen Extraction Fraction in Carotid Occlusive Disease: Influence of Cerebral Autoregulation and Cerebral Blood Volume
Source: PLoS One. 2016 Aug 25;11(8):e0161408. doi: 10.1371/journal.pone.0161408 (PMC4999181; doi:10.1371/journal.pone.0161408)
Supplement: S2 Table — Due to the rather small differences in rCBV-values between the hemispheres, these data are presented with two decimals. In patient 5 and patient 14 no MTT-delay was detected. q: quantitative; rCBV: relative cerebral blood volume; TTP: time-to-peak; MTT: mean transit time. *Baseline absolute values of qT2’ for the entire TTP-and MTT-delayed areas have already been presented in a previous publication (Seiler A, Jurcoane A, Magerkurth J, Wagner M, Hattingen E, Deichmann R et al. T2' imaging within perfusion-restricted tissue in high-grade occlusive carotid disease. Stroke. 2012;43:1831–1836). (DOCX) [file pone.0161408.s004.docx]

|  | T2’ TTP hypoperfusion*  [ms] | |  | rCBV TTP hypoperfusion  [ml/100 g] | |  | T2’ MTT hypoperfusion*  [ms] | |  | rCBV MTT hypoperfusion  [ml/100 g] | |  |
| --- | --- | --- | --- | --- | --- | --- | --- | --- | --- | --- | --- | --- |
| Patient No. | Affected hemisphere | Unaffected hemisphere | Ratio | Affected hemisphere | Unaffected hemisphere | Ratio | Affected hemisphere | Unaffected hemisphere | Ratio | Affected hemisphere | Unaffected hemisphere | Ratio |
| 1 | 118 | 123 | 0.96 | 4.42 | 4.52 | 0.98 | 119 | 125 | 0.95 | 4.66 | 4.71 | 0.99 |
| 2 | 120 | 108 | 1.11 | 3.98 | 4.09 | 0.97 | 114 | 113 | 1.01 | 4.57 | 4.53 | 1.01 |
| 3 | 120 | 119 | 1.01 | 6.45 | 7 | 0.92 | 118 | 122 | 0.97 | 6.95 | 7.34 | 0.95 |
| 4 | 106 | 109 | 0.97 | 7.3 | 7.36 | 0.99 | 105 | 109 | 0.96 | 7.01 | 7.03 | 1.00 |
| 5 | 111 | 106 | 1.05 | 4.39 | 5.72 | 0.77 | n.d. | --- | --- | --- | --- | --- |
| 6 | 136 | 138 | 0.99 | 15.33 | 16.78 | 0.91 | 134 | 134 | 1.00 | 14.28 | 17.14 | 0.83 |
| 7 | 118 | 130 | 0.91 | 6.19 | 6.48 | 0.96 | 117 | 130 | 0.90 | 6.32 | 6.39 | 0.99 |
| 8 | 119 | 139 | 0.86 | 10.05 | 10.08 | 1.00 | 120 | 137 | 0.88 | 10.62 | 10.39 | 1.02 |
| 9 | 96 | 147 | 0.65 | 7.38 | 8.01 | 0.92 | 97 | 143 | 0.68 | 7.76 | 8.31 | 0.93 |
| 10 | 119 | 136 | 0.88 | 6.63 | 6.85 | 0.97 | 117 | 135 | 0.87 | 7.23 | 7.05 | 1.03 |
| 11 | 122 | 129 | 0.95 | 4.72 | 5.67 | 0.83 | 115 | 132 | 0.87 | 4.74 | 5.09 | 0.93 |
| 12 | 105 | 116 | 0.91 | 8.37 | 8.67 | 0.97 | 105 | 117 | 0.90 | 8.65 | 8.56 | 1.01 |
| 13 | 108 | 130 | 0.83 | 4.08 | 4.17 | 0.98 | 107 | 135 | 0.79 | 4.44 | 4.31 | 1.03 |
| 14 | 114 | 126 | 0.91 | 10.54 | 10.04 | 1.05 | n.d. | --- | --- | --- | --- | --- |
| 15 | 122 | 131 | 0.93 | 7.54 | 7.44 | 1.01 | 126 | 134 | 0.94 | 7.25 | 7.03 | 1.03 |
| 16 | 117 | 123 | 0.95 | 10.61 | 11.51 | 0.92 | 115 | 123 | 0.93 | 11.83 | 11.95 | 0.99 |
| Median (25^th^; 75^th^ percentile) | 118 (108.75;120) | 127.5 (116.75;134.75) | 0.93 (0.88; 0.99) | 6.97 (4.5; 9.63) | 7.18 (5.68; 9.7) | 0.97 (0.92;0.99) | 116 (106.5;119.25) | 131 (120.75;135) | 0.92 (0.87;0.96) | 7.12 (4.72; 9.14) | 7.04 (5; 9.01) | 1 (0.95;1.02) |

**S2 Table**. Individual patient data: median values and hemispheric ratios for T2’ and rCBV in areas with any TTP- and MTT-delay (> 0 seconds).
